# Species on the move around the Australian coastline: A continental‐scale review of climate‐driven species redistribution in marine systems

**DOI:** 10.1111/gcb.15634

**Published:** 2021-05-07

**Authors:** Connor R. Gervais, Curtis Champion, Gretta T. Pecl

**Affiliations:** ^1^ Department of Biological Sciences Macquarie University Sydney NSW Australia; ^2^ Fisheries Research NSW Department of Primary Industries Coffs Harbour NSW Australia; ^3^ Southern Cross University National Marine Science Centre Coffs Harbour NSW Australia; ^4^ Institute for Marine and Antarctic Studies University of Tasmania Hobart Tas Australia; ^5^ Centre for Marine Socioecology University of Tasmania Hobart Tas Australia

**Keywords:** citizen science, climate change, ecosystem reorganization, historical data, ocean warming, range contraction, range extension, range shift

## Abstract

Climate‐driven changes in the distribution of species are a pervasive and accelerating impact of climate change, and despite increasing research effort in this rapidly emerging field, much remains unknown or poorly understood. We lack a holistic understanding of patterns and processes at local, regional and global scales, with detailed explorations of range shifts in the southern hemisphere particularly under‐represented. Australian waters encompass the world's third largest marine jurisdiction, extending from tropical to sub‐Antarctic climate zones, and have waters warming at rates twice the global average in the north and two to four times in the south. Here, we report the results of a multi‐taxon continent‐wide review describing observed and predicted species redistribution around the Australian coastline, and highlight critical gaps in knowledge impeding our understanding of, and response to, these considerable changes. Since range shifts were first reported in the region in 2003, 198 species from nine Phyla have been documented shifting their distribution, 87.3% of which are shifting poleward. However, there is little standardization of methods or metrics reported in observed or predicted shifts, and both are hindered by a lack of baseline data. Our results demonstrate the importance of historical data sets and underwater visual surveys, and also highlight that approximately one‐fifth of studies incorporated citizen science. These findings emphasize the important role the public has had, and can continue to play, in understanding the impact of climate change. Most documented shifts are of coastal fish species in sub‐tropical and temperate systems, while tropical systems in general were poorly explored. Moreover, most distributional changes are only described at the poleward boundary, with few studies considering changes at the warmer, equatorward range limit. Through identifying knowledge gaps and research limitations, this review highlights future opportunities for strategic research effort to improve the representation of Australian marine species and systems in climate‐impact research.

## Graphical abstract

With the continued rise in sea surface temperature, species redistributions are becoming more commonplace around the world. Around Australia, at least 198 marine species, from 9 phyla, are redistributing their distributions, 87% of which are shifting poleward, in accordance with climate change.

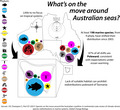

## 1 INTRODUCTION

Marine systems are at the forefront of climate‐driven environmental change, with the ocean taking up more than 90% of the additional heat trapped in the atmosphere, resulting in rapid warming and an increase in the frequency and intensity of marine heatwaves (IPCC, 2020). Already, some marine species are living under conditions at or near their thermal limits and small increases in ocean temperatures will thus result in a decline in the performance and health of these species (Pörtner & Farrell, 2008). However, these changes are also occurring alongside an acceleration in the extent, intensity and diversity of human uses of marine systems (Jouffray et al., 2020), and an increase in the cumulative human impacts on marine ecosystems across most of the ocean (Halpern et al., 2019). Biological responses are often negatively linked with these concomitant, anthropogenic stressors (Poloczanska et al., 2013) and already we are observing alterations in marine systems from individual species up to entire ecosystems (Pinsky et al., 2020).

One of the most pervasive responses to climate‐driven warming is the redistribution of life on Earth (Lenoir et al., 2020), with wide‐ranging implications for human well‐being, ecosystem function and the climate itself (Pecl et al., 2017). There is, however, great taxonomic variation in the pace and magnitude of this shifting geography of life, along with regional and system‐level differences. In the ocean, there are fewer barriers to movement than on land, and the thermal safety margins of marine species are narrower than those of terrestrial species (Pinsky et al., 2019), meaning that marine taxa are better equipped to more closely track local shifts in temperature isotherms than their terrestrial counterparts (Pinsky et al., 2013). As such, marine ectotherms are both more sensitive to warming temperatures than species on land, and more able to respond through shifts in geographical distribution (Sunday et al., 2012). Indeed, marine species are shifting their distributions poleward on average six times faster than terrestrial species (Lenoir et al., 2020), as well as displaying nearly double the rate of extirpations (Pinsky et al., 2019). However, few marine species, even in very fast‐warming regions, are completely keeping pace with climate (Fredston‐Hermann et al., 2020). This is likely because the location of range edges is a function of the interplay between biological and physical factors (Baselga et al., 2012), rather than climate alone.

In addition to local and regional climate, species distributional responses are also a result of species‐specific physiological, behavioural, ecological and evolutionary responses (William et al., 2009), along with biological interactions (Ling, 2008; Wisz et al., 2013), and the extent and nature of additional stressors (Zhang et al., 2020). In general, at warmer range edges where elevated temperatures exceed physiological tolerances, species distribution edges are typically contracting, while at cooler range edges, where there is an increasing availability of suitable conditions, species distributions are typically extending. However, the rates of species redistributions may be modulated by factors such as habitat availability (Feary et al., 2014; Nay et al., 2020) and predator–prey interactions (Kordas et al., 2011). Furthermore, there is some evidence of ‘invasional meltdown’ at species range edges where the presence of range shifting species facilitates the establishment of additional climate‐driven species arriving within the same region (Bates et al., 2017; Ling et al., 2018).

Climate‐driven redistribution of species is leading to wide‐spread community restructuring and the creation of ‘novel’ ecosystems, particularly in the ocean, with species richness increasing with warming in most regions (Antão et al., 2020). In general, climate‐driven range extensions are five times faster than range contractions in the ocean (Poloczanska et al., 2013), so for many geographical locations, species gains are outpacing species losses. The extension or contraction of structure‐forming species can have strong cascading flow‐on effects for the broader ecosystem, resulting from the gain or loss of these critical habitats. Moreover, changes in the distribution of species with particularly strong influence on the ecosystem have the potential to alter ecological network structure to the extent of leading to new ecosystem regimes (Johnson et al., 2011), and some species can have ecological impacts equivalent to invasive species (Ling, 2008).

Despite the pervasive nature of climate‐driven changes in species distribution, our detection, description and understanding of these changes is far from comprehensive, and substantial gaps remain. Our current understanding of range shifts globally is biased towards more charismatic species, and the most developed regions of the Northern Hemisphere (Lenoir et al., 2020). Detailed explorations of species range shifts in the Southern Hemisphere are under‐represented, and as such are in part limiting our potential to generate the process‐based understanding needed for stronger predictive capacity of future shifts in species distributions (Twiname et al., 2020). The implications of widespread changes in species distributions are substantial for coupled‐socioecological systems through, for example, alterations to fishing and tourism opportunities (Champion et al., 2019), novel threats to aquaculture (e.g. new diseases) and changes to conservation and spatial planning paradigms (Scheffers & Pecl, 2019). Identifying and addressing gaps in our understanding of current and projected species range shifts will facilitate the development of effective adaptation responses required to minimize negative impacts and maximize opportunities associated with climate‐driven species redistributions (Bonebrake et al., 2017; Pecl et al., 2017).

Australia's marine environment encompasses 13.86 million square kilometres, making it the world's third largest marine jurisdiction. A defining characteristic of Australia's approximately 50,000 km long coastline is that it encompasses tropical to subantarctic climate zones and ecosystems, and the oceanographic context is unique in that both western (Leeuwin current) and eastern (East Australian Current) Australian coastlines are dominated by poleward flowing boundary currents (Suthers et al., 2011; Waite et al., 2007). These oceanographic features transport warm water from tropical to temperate regions and underpin the distributions and seasonal migration of numerous marine species (Brodie et al., 2015; Malcolm & Scott, 2017; Ramos et al., 2018).

**FIGURE 1:**
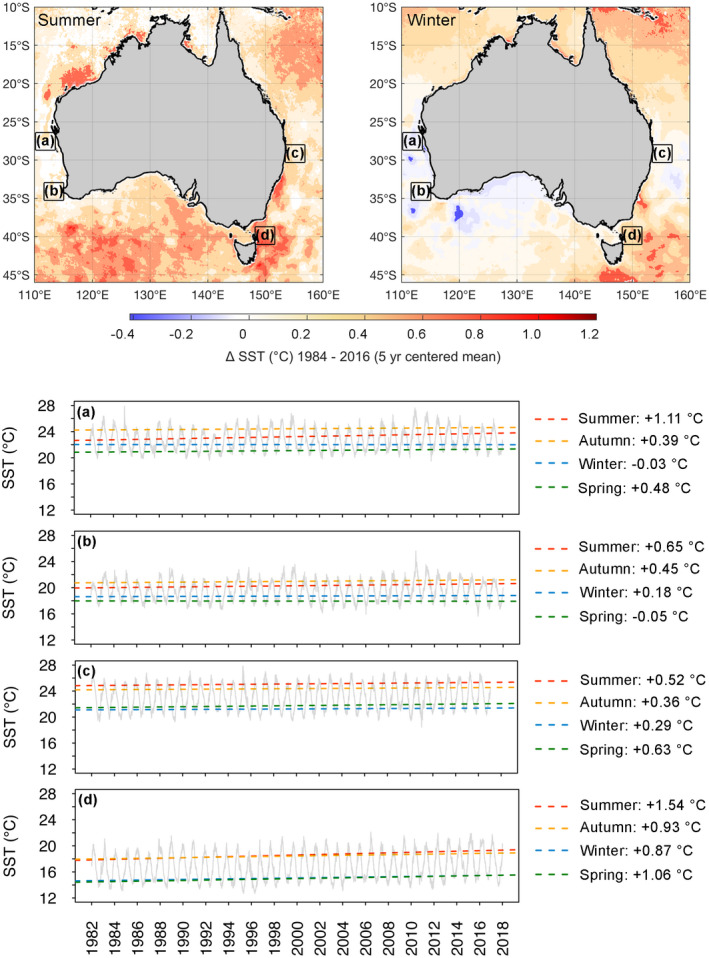
Changes in mean summer and winter sea surface temperature surrounding Australia from 1982 to 2018 based on 5‐year means centred on 1984 and 2016 (upper panels) and corresponding time‐series for four geographically explicit regions (a–d) of Australia's surrounding ocean. Trends in sea surface temperature data are presented for summer (red lines), autumn (orange lines), winter (blue lines) and spring (green lines) annual averages from each region. Data source: Daily global sea surface temperature reprocessed (level 4) from the Operational SST and Ice Analysis system (OSTIA), downloaded from the Copernicus Marine Environment Monitoring Service (https://marine.copernicus.eu; product #010_011)

Extensive climate‐driven environmental change is now apparent throughout Australian marine systems (Hobday & Pecl, 2014; Oliver et al., 2017; Pearce & Feng, 2013). The East Australian Current has extended a further 350 km southwards over the last 70 years (Pecl et al., 2009; Ridgway, 2007), and both the south‐east and south‐west regions of Australia are recognized as ocean warming ‘hotspots’, in the top 10% for rates of temperature increase globally (Hobday & Pecl, 2014). Moreover, the rate of ocean warming around Australia has accelerated in recent decades (Figure 1), with the greatest increases in sea surface temperature (SST) occurring off the south‐eastern coastline. Increasing sea surface temperatures have been exacerbated by marine heatwaves off south‐east Australia in 2015/2016 and 2017/2018, which have lasted up to 251 days and reached a peak intensity of ~3°C above the long‐term climatology (Oliver et al., 2017; Perkins‐Kirkpatrick et al., 2019).

Extreme marine heatwaves off the south‐west Australian coast in 2011 led to a 100 km range contraction of kelp forests (Wernberg et al., 2016), and changes in regional fish assemblages (Shalders et al., 2018; Teagle et al., 2018). Indeed, extreme climatic events in Australia from 2011 to 2017 resulted in abrupt and extensive mortality of key habitat‐forming organisms, including corals, kelps, seagrasses and mangroves, along >45% of the continental coastline of Australia (Babcock et al., 2019). Back‐to‐back regional‐scale bleaching events across much of the Great Barrier Reef in 2016 and 2017, followed by a third major bleaching event in 2020, have led to extensive reductions in live coral cover (Hughes et al., 2018). These extreme climatic events are occurring with increasing frequency and intensity and are facilitating changes that are ultimately altering species community structure and ecosystem services at both local and regional scales (Smale et al., 2019). Australian waters are particularly vulnerable to potential biodiversity loss due to the high rate of temperature change, high number of endemic species and at the southern continental limits, no continental shelf habitats further south for climate shifting species to move into. However, a detailed and comprehensive assessment of available evidence regarding species redistribution along the entire Australian coastline has not been undertaken.

Here, we conduct a multi‐taxon review to develop a continent‐wide synthesis of the current trends in marine species redistribution around Australia—both observed and predicted. At a continent‐wide scale, the marine seascape of Australia is varied and encompasses a large latitudinal breadth situated under a common jurisdictional legislature. We aim to highlight gaps in our knowledge of species redistribution around the Australian coastline that are presently limiting our capacity to develop strategic options for prioritization of future research programs. Moreover, much of our current understanding of range shifts stems from single species studies (e.g. Ramos et al., 2018), regional‐scale analyses (e.g. Sunday et al., 2015) or global‐level assessments (e.g. Lenoir et al., 2020), and a continental‐scale assessment may help inform future redistribution studies elsewhere.

## 2 SYSTEMATIC LITERATURE SEARCH

We undertook a systematic search for climate‐driven marine range shifts from Australia using the Web of Science database. Despite the relatively nascent interest in range shifts, there are already a number of emerging patterns in how and where range shifts are reported which can over‐ or under‐represent the true nature of range shifts. Understanding these variations can provide direction for future studies and allow more efficient, targeted research to address areas where data are lacking. Therefore, we specifically aimed to identify the methodological approaches used to detect range shifts (Section 4), identify spatial variability and regional focus of range shift studies (Section 5), and generate a continent‐wide assessment of the nature of changes in the distribution of marine species around Australia (Section 6).

Search terms were designed to capture all available literature pertaining to Australian marine range shifts and climate change. Given the numerous terminologies and classifications describing species range shifts, the marine environment and climate change in the literature, multiple search terms were required to assemble a comprehensive database of published research. The following terms were used to identify, refine and collate available literature:

Australia* AND ((Marine*) OR (Ocean) OR (Coral reef*) OR (Kelp forest) OR (Sea*grass) OR ((Tropic*) OR (Temperate) OR (Sub*tropic*))) AND ((Range shift) OR (Distribution shift) OR (Range contraction) OR (Range expansion) OR (Range extension)) AND ((Climate warming) OR (Climate change*) OR (Temperature increase*) OR (Precipitation change*) OR (Acidification) OR (Decreas* pH))

​

As range shifts are dynamic, occurring progressively over time and often through distinct transitional stages from early stages of arrival/departure to the establishment/extirpation of self‐sustaining populations (Bates et al., 2014), only range shifts that were considered to be in stages of ‘population increases/decreases’ or ‘species persistence/local extinction’ (i.e. range shift stages 2 and 3, respectively, as per Bates et al., 2014) were included. With respect to range extensions, papers indicative of non‐established species or populations (e.g. vagrant, juvenile‐only observations, non‐overwintering or transient) were excluded (e.g. Booth et al., 2007; Nimbs et al., 2016). Additionally, range shift studies often rely on baseline data to support the evidence of contemporary range shifts; however, there can be inherent biases within baseline data, specifically collection biases or low sampling effort which may hinder accurate representation of a species historic distribution (see Przeslawski et al., 2012). Therefore, papers indicating that observed range extensions were likely to be a function of increased sampling effort, rather than a climate‐mediated shift in a species’ distribution (e.g. Nimbs et al., 2015; Schoeman et al., 2015), were also excluded.

Relevant papers were read in full to collate information pertaining to species range shifts, and their references were checked to ensure additional literature was not overlooked. In this review, range shift literature falls into one of two categories, observed range shifts or predicted (historical and future) range shifts. Papers that documented observed range shifts were analysed to identify trends related to methodological approaches, spatial patterns and biases and species range shift trajectories (i.e. range contractions or extensions at equatorward or poleward range edges). Papers concerning predicted range shifts were analysed for trends related to modelling approaches, prediction time scales and species of interest.

## 3 TEMPORAL TRENDS IN RESEARCH

**FIGURE 2:**
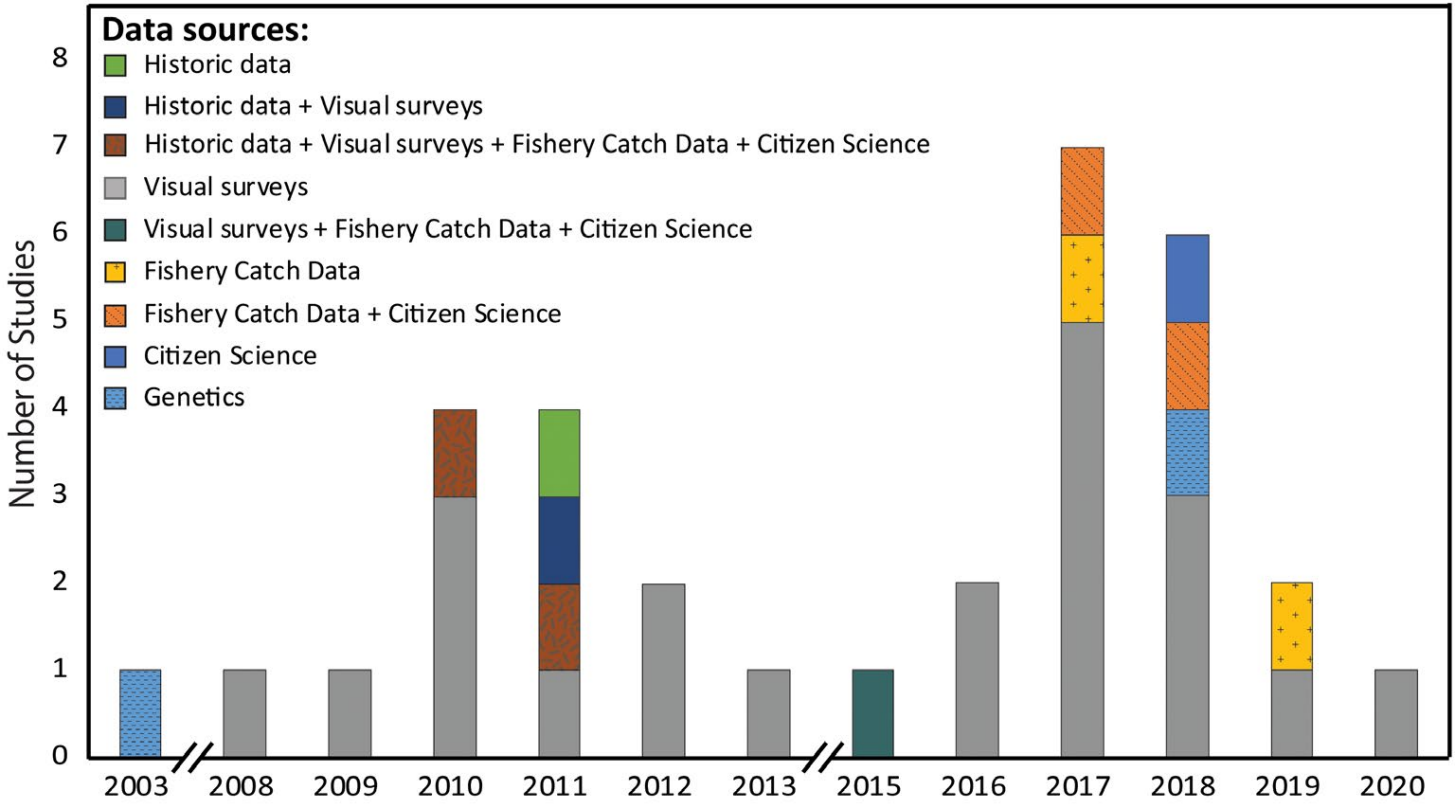
Temporal trends in observed Australian marine range shift literature. Bars represent the number of publications per year and colours represent the varied data sources that studies have used to observe and verify species range shifts

Our literature search returned 454 papers and the abstracts of each were read to establish relevance. Of the returned papers, 49 (11.2% of the total returned papers) met the review criteria (Table S1). Research on observed range shifts accounted for double the number of papers (*n* = 33; encompassing 198 marine species; Table S2) than those utilizing quantitative prediction methods for determining range shifts (*n* = 16, encompassing 102 marine species, Table S3). Prior to 2003, there was no published research concerning marine range shifts in Australia (observed or predicted), despite research regarding marine climate change indicating that changes in species distributions were likely (Hughes, 2000, 2003) and research in other parts of the world documenting range shifts prior to this time (Kennedy, 1990; Parmesan & Yohe, 2003). Even so, published research from Australian marine systems was largely lacking until 2008, with only one previous study observing any shifts in distribution (Gopurenko et al., 2003). Over time there has been no consistent trend in the number of publications per year (Figure 2); however, the number of studies in Australia has increased.

## 4 APPROACHES USED TO DETECT AND PREDICT RANGE SHIFTS

Many different general approaches and data sets are used to detect and subsequently verify range shifts (Booth et al., 2011; Twiname et al., 2020). Given the variability and sometimes opportunistic degree to which data have been collected, there has been little standardization across studies or within general approaches undertaken. Booth et al. (2011) defined many of the approaches that can and have been used to identify range extensions in fish, of which most are applicable across taxa. Within the 33 papers documenting observed marine range shifts around Australia, there are a number of approaches that have been employed (Figure 2; Table 1).

**TABLE 1:** Variation in methodological measures and temporal scales presented in the literature reporting observed and predictive range shifts. Please note that the total sum of papers can be greater than the 33 observation‐based or 16 predictive studies, as some papers report multiple metrics

	Number of publications	Per cent of publications (from 33 observation‐based or 16 predictive publications)
Observed measures		
Species abundance	20	60.6
Species presence	18	54.5
Species presence/absence	7	21.2
Genetic connectivity	2	6.1
Data temporal scale		
Time‐series (>10 years)	7	21.2
Time‐series (>5 years)	1	3.0
Time‐series (<10 years)	3	9.1
Time‐series (<5 years)	2	6.1
Before/after surveys (>10 years)	7	21.2
Before/after surveys (<10 years)	5	15.2
Opportunistic (>10 years)	3	9.1
Single observation period (against historical distribution)	3	9.1
Predictive measures		
Centroid (area of greatest abundance)	8	50
Future distribution	4	25
Phenology	1	6.25
Establishment probability	1	6.25
Habitat area loss	1	6.25
Change in distributional limit	1	6.25

An accurate and complete baseline distribution of a species is often needed to appropriately assess distributional shifts over time (Booth et al., 2011), unless using a qualitative assessment approach that considers the certainty with which the baseline distribution was known (e.g. Robinson, Gledhill, et al., 2015). In Australia, this baseline information is often relatively limited, and likely contributes to the use of specific approaches, depending on the target species (or ecosystem; Booth et al., 2011). For instance, historical fishery‐dependent catch data have been collected continuously and may provide information regarding the target species, while information regarding non‐target species—or any species of negligible commercial or ecological value—often lacks similar data quantity and quality. Thus, the available baseline distributional studies (or lack thereof) often influence the approaches used to subsequently detect contemporary range shifts.

### 4.1 Historical data

The importance of historical data sets cannot be overstated, as contemporary studies rely on at least some understanding of past distributions. While limited, in Australia, there are three main sources of historical data sets that have been used in this manner: fishery‐dependent catch records, historical biodiversity surveys and museum or herbarium collections (Figure 2). This range of sources is invaluable given that these encompass a large range of species and taxonomic groups. Specifically, historical data sets—analysed in four papers (Last et al., 2010; Pitt et al., 2010; Poloczanska et al., 2007; Wernberg et al., 2011)—provide the necessary historical range limits to verify shifting distributions (extensions or contractions) of 159 Australian marine species (67.7% of total species shifts captured in this study).

Given that historical sources were typically not collected for the purpose of monitoring species range shifts, there can be limitations in their suitability for detecting distributional shifts (Pitt et al., 2010; Poloczanska et al., 2007; Tingley & Beissinger, 2009). Inherent biases represented within these sources (e.g. different survey methods or patchy sampling effort and spatial coverage) confound the capacity to accurately quantify species distributions (Booth et al., 2011; Brown et al., 2016). For example, sampling bias and unequal spatiotemporal sampling effort in Southern Australia museum collections impeded the ability to confidently identify shifts in Australian mollusc distributions, even among sites and species associated with the greatest sampling effort (Przeslawski et al., 2012). While there are limitations to the use of historic collections, rigorous selection criteria and supplementary surveys can minimize these limitations, allowing for the use of these valuable data sets to detect shifting species distributions, particularly when existing baseline information are limited (Poloczanska et al., 2007; Tingley & Beissinger, 2009).

### 4.2 Underwater visual surveys

While historical data provide baselines for numerous species, underwater visual surveys (UVS) are the most common method employed to document and/or confirm species range shifts. Of the 33 observational range shift studies from Australia, 25 (75.7%) incorporate UVS methodologies and of those 21 rely on UVS as the sole source to document the range shifts. There is, however, a large degree of variation in the specific protocols and analyses used, from historical biodiversity surveys (e.g. Last et al., 2010) to contemporary studies highlighting the acute and chronic impacts of extreme ocean events, specifically marine heatwaves (e.g. Smale et al., 2017), through to opportunistic species observations (Baird et al., 2012; Table 1). In addition, the metrics reported vary from species presence (which can support only range extensions), species presence and/or absence (which can demonstrate both range extensions and contractions) or species abundance (which can provide the best resolution for the stage of a species redistribution). With such varied survey protocols, the most robust and accurate studies are those which undertake repeated UVS across a range of sites over regular time scales (Tingley & Beissinger, 2009).

Without adequate time‐series data, persistent temporal trends in species distributional limits—especially those following extreme climatic events—can be overestimated, or conversely, overlooked (Fredston‐Hermann et al., 2020). For example, Cure et al. (2015), conducted UVS in 2013 and reported a 2011 marine heatwave event facilitated an above average recruitment of a sub‐tropical wrasse (*Choerodon rubescens*) at its most southern distribution. Additional surveys were conducted in 2014–2015 in which the density of this species, while still very much present in the area, was markedly lower than in 2013 (Cure et al., 2018). Follow‐up surveys are necessary to assess if the trajectory of species redistributions (both extensions and extirpations) continues, especially following extreme events such as marine heatwaves. Short time‐frame UVS—such as those opportunistically recording out‐of‐range observations or undertaking acute before/after impact surveys—are valuable for detecting range shifts, but without careful consideration and experimental design are sometimes unable to verify species range shifts or establish long‐term residency trends.

Longer‐term studies provide a more accurate and robust assessment of species redistributions, although survey data analysed within these can be associated with relatively coarse temporal resolutions due to a suite of limitations, including accessing and maintaining consistent long‐term funding.

### 4.3 Citizen science

To overcome some of the logistical and monetary limitations that can inhibit career scientists from conducting rigorous long‐term biodiversity and abundance monitoring, non‐profit citizen science programs have arisen to fill the role. Given the number of people observing and documenting wildlife daily and the knowledge that some local communities have regarding natural patterns, the potential for citizen scientists to detect range shifting species, specifically range extensions, is substantial. As local communities are often the first to detect out‐of‐range species, citizen science projects are essential to identify the onset of shifts within ecosystems and facilitate early management if needed. Across ecology, citizen science efforts have been useful in detecting patterns in phenology, changes in species richness and community composition, as well as distributional shifts (Dickinson et al., 2010). Indeed, within Australia, 6 of 33 range shift studies incorporate citizen science observations to supplement additional data sets (e.g. historical data or underwater visual surveys). Australia has a myriad of established citizen science databases (e.g. Redmap Australia, iNaturalist, Living Atlas of Australia, Reef Life Survey) in which the public submit observations of species, some of which, depending on the program, are verified by leading scientific experts. Among these, Redmap Australia (range extension database and mapping project, www.redmap.org.au) was specifically developed to detect observations of Australian marine species observed or caught outside of their known range, as ‘an early indication’ of species that may be shifting (Pecl, Barry, et al., 2014; Pecl, Stuart‐Smith, et al., 2019). Furthermore, Reef Life Survey (RLS) is an Australian organization that trains recreational SCUBA divers in the use of a standardized method for surveying the biodiversity of rocky and coral reefs (Edgar & Stuart‐Smith, 2014; www.reeflifesurvey.com). The design and establishment of standardized citizen science programs, such as RLS, allows for consistent data collection across larger spatial scales and finer temporal resolutions, which can facilitate robust range shift analyses. Citizen scientists can provide information over large spatial and temporal scales, and from regions or at times not typically surveyed or accessed by scientists. Such programs can also be useful in elevating public awareness of climate change and range shifting species (e.g. see Nursey‐Bray et al., 2018, for a formal evaluation of Redmap Australia in this regard). However, it should be noted that citizen science programs also need long‐term financial security to operate successfully (Pecl, Stuart‐Smith, et al., 2019).

### 4.4 Genetic sampling

A relatively novel approach to documenting and verifying range shifts has been through the use of genetic sampling. This approach focuses on identifying population connectivity, genetic structure and diversity of a species genotype across its historical and purported novel distribution (Ramos et al., 2018). Understanding population structure and genetic diversity has been useful in identifying the source population(s) of range expanding species (Gopurenko et al., 2003), as well as predicting the capacity for species to arrive and persist in new locations (Ramos et al., 2018). Species with low dispersal capacities may exhibit genetic bottleneck effects and low genetic variation within new range areas, which is likely to impede the establishment of self‐sustaining populations in novel regions (Excoffier et al., 2009). In contrast, species that exhibit high dispersal and high genetic diversity at their range edges are more likely to persist and extend into new areas (Ramos et al., 2018). Three Australian species, *Octopus tetricus*, *Centrostephanus rodgersii* and *Scylla serrata*, are exhibiting rapid range extensions with no evidence of low genetic diversity at the leading range edge (Banks et al., 2010; Gopurenko et al., 2003; Ramos et al., 2018), which has been attributed to the broad dispersal of larvae throughout their entire distribution.

Environmental DNA (i.e. DNA that can be collected from environmental samples such as sediments or water) is becoming increasingly used to detect the presence of rare or invasive species in an area (Bohmann et al., 2014; Boussarie et al., 2018). While little to no published literature currently uses eDNA to detect range shifts as a consequence of climate change (in Australia or elsewhere), this non‐invasive methodology can quickly and efficiently sample a large number of species across broad geographical regions. Already, eDNA has been used to detect marine species rarely observed using traditional survey methods as well as construct contemporary community structure baselines (Boussarie et al., 2018; Djurhuus et al., 2020; Miya et al., 2015; Thomsen et al., 2016). For example, following the 2011 marine heatwave event in Western Australia, eDNA assays reported changes in copepod diversity and richness (Berry et al., 2019). As with most methodologies, there are caveats in using this technique that need to be considered and it may not be appropriate for all aspects of detecting range shifts (specifically range contractions; see Roussel et al., 2015). However, with further refinement, eDNA sampling approaches are likely to be incorporated into future range shift studies.

### 4.5 Predictive modelling

Predicting range shifts over historical and future periods is dependent on accurately estimating species distributions at multiple time points. Our literature analysis identified 16 manuscripts that quantitatively predict changes in the distribution or range size of 100 marine species around the Australian coastline. While multiple methods for developing species distribution models (SDMs) can be used to predict range shifts, including grid‐based mapping, convex hull, kriging and hybrid approaches (Yalcin & Leroux, 2017), correlative approaches have been extensively applied within the Australian marine context (i.e. 11 of 16 predictive modelling studies). Subsequently, range shifts in 74 species have been determined by correlating species occurrence or abundance data with environmental predictors of species distributions, such as sea surface temperature (Robinson et al., 2011), and quantifying geographical shifts in the location of species environmental habitat preferences. It is important to recognize that range shifts predicted in this way are not necessarily representative of species’ realized distributions directly, but rather reflect the distribution of species’ environmental habitat preferences. Furthermore, range shift analyses based on correlative relationships between species and their environment are underpinned by the key assumption that historical species responses will remain consistent under current and future, potentially novel, environmental conditions. Nevertheless, correlative approaches allow for predictions of species distributions at spatiotemporal resolutions that environmental covariates are available (e.g. daily to seasonal), facilitating range shift analyses for Australian marine species that are not associated with structured survey data (Champion et al., 2018; Hill et al., 2016).

Our literature analysis identified a bias towards coastal‐pelagic fishes, which were the focus of 10 of 16 predictive range shift studies. Two explanations for this apparent bias include (1) that fishery‐dependent data sets containing information about the location of coastal‐pelagic fishes around Australia over long periods of time (i.e. 20 years) are available (e.g. Brodie et al., 2015), and (2) that offshore habitats occupied by coastal‐pelagic fishes are well‐suited for analyses that utilize satellite‐derived oceanographic covariates to quantify and assess for spatial shifts in species environmental habitat preferences and use these habitats as proxies for species distributions. Subsequently, species distribution models that quantify oceanographic habitat suitability have underpinned range shift analyses for various commercially and recreationally important coastal‐pelagic fishes from Australia, including black marlin (*Istiompax indica*; Hill et al., 2016), yellowtail kingfish (*Seriola lalandi*; Champion et al., 2018), yellowfin tuna (*Thunnus albacares*; Dell et al., 2015), southern bluefin tuna (*Thunnus thynnus*; Robinson, Hobday, et al., 2015) and dolphinfish (*Coryphaena hippurus*; Hobday, 2010).

Aspects of predictive modelling that can affect estimates of species range shifts include the length of time‐series used to quantify rates of redistribution (Fredston‐Hermann et al., 2020), whether environmental data used to estimate species distributions have been observed or modelled, the component of species ranges analysed (e.g. leading or trailing edges; Champion et al., 2018) and the number and combination of environmental covariates in SDMs (Brodie et al., 2019; McHenry et al., 2019). For example, Hobday (2010) and Robinson, Hobday, et al. (2015) utilized modelled future climate data without bias correction to predict species redistributions, while Hill et al. (2016) and Champion et al. (2018) utilized historical satellite observations. Furthermore, Payne et al. (2018) show that abundance and performance data can be correlated with temperature to predict the redistribution of thermal habitat for tiger sharks (*Galeocerdo cuvier*) off eastern Australia, which differs from other studies that rely on species occurrence data to quantify environment habitat preferences and estimate range shifts (Hill et al., 2016; Hobday, 2010). Subsequently, this review does not attempt to compare predicted rates of change in the distributions of Australian marine taxa, and we instead recommend that results from predictive analyses should be interpreted with respect to the methods used to derive them. Recent efforts to identify relevant methodologies for studying the various stages and processes involved in the climate‐driven redistribution of marine species appear promising for increasing the comparability of range shift analyses. For example, Twiname et al. (2020) provide a synthesis of the laboratory, field and modelling approaches appropriate for studying redistribution related processes at individual, population and community levels in marine systems. Frameworks such as these have the potential to improve the capacity for effective synthesis and comparison of research being undertaken in this rapidly expanding field, ultimately facilitating the identification of emerging trends and knowledge gaps.

Our analysis of literature predicting marine range shifts around Australia identified considerable scope for improving the representation of species whose distributions are poorly predicted by remotely sensed environmental data (e.g. small tropical fishes and intertidal invertebrates), and how predictions can be tailored for stakeholders. First, while standardized methods are desirable for increasing the comparability of range shifts predicted using SDMs, alternative methods are still required to increase the relatively low representation (~38%) of species other than coastal‐pelagic fishes in Australian marine range shift analyses. For example, a novel morphological niche analysis undertaken by Smith et al. (2016) was used to predict the probability of 11 vagrant tropical fishes establishing beyond the current poleward limit of their east Australian distributions. This analysis also quantified morphological niches for 110 fishes from the likely recipient assemblage, highlighting the utility of this method for predicting the likelihood of successful range extensions in a large number of species. Second, predictions of past and future climate‐driven range shifts can produce metrics of direct relevance to stakeholders that are potentially useful to aid decision‐making (Hobday et al., 2016), yet these attributes were only identified in two quantitative studies reviewed (Champion et al., 2019; Jacups, 2010). For example, Champion et al. (2019) predicted climate‐driven changes in the temporal persistence (i.e. months per year) of suitable environmental habitat for yellowtail kingfish (*S*. *lalandi*) within spatially explicit regions of eastern Australia, and linked this metric with social and economic fishing opportunity. Similarly, Jacups (2010) utilized the correlation between sea surface temperature and box‐jellyfish (*C*. *fleckeri*) stings in the Northern Territory of Australia to predict an increase in the annual duration of the *C*. *fleckeri* stinger season in this region under future ocean warming. These examples demonstrate that the temporal persistence of species preferred environmental conditions is a stakeholder‐relevant metric that may be quantified in future research aiming to support the development of climate change adaptation options for range shifting species.

## 5 SPATIAL VARIABILITY IN RANGE SHIFT OBSERVATIONS

Unsurprisingly, highly populated areas are among the most easily accessible for undertaking research, reflected in Australia as a focus on Tasmania, the south‐east coastline (southern Queensland to Victoria) and the south‐west coastline (southern Western Australia; Hugo, 2002). However, this presents a bias both in terms of the species observed to be undergoing climate‐driven redistributions and the latitudinal location of species range edges captured in scientific surveys. A key consideration when making geographical comparisons of marine species distributions is the orientation of coastal environments with respect to the direction of climate change. North‐south orientated coastlines, including eastern and western Australian coasts, are generally aligned in parallel with the direction of climate change, whereas marine species off east‐west orientated coastlines are likely to be exposed to slower rates of ocean warming which may mediate climate‐driven range shifts in these regions. This may explain why the vast majority of range shifts in marine species from Australia have been documented along western, south‐eastern and Tasmania coastlines. Moreover, there is a greater quantity of climate change related marine research being published from both south‐east and south‐west Australia, particularly for commercially valuable species (Fogarty et al., 2019). To date, only one species, *Chironex fleckeri*, has been observed undergoing a possible range shift in the Northern Territory, a region of the Australian coastline other than those aforementioned (Jacups, 2010). With increases in average sea surface temperatures, the *C*. *fleckeri* stinger season in Darwin (Northern Territory) is likely increasing. As such, changes in phenology may be more indicative of climate‐mediated impacts along east‐west running coastlines, where no latitudinal gradients occur; however, phenology‐focused studies on marine species are relatively few (Poloczanska et al., 2013).

**FIGURE 3:**
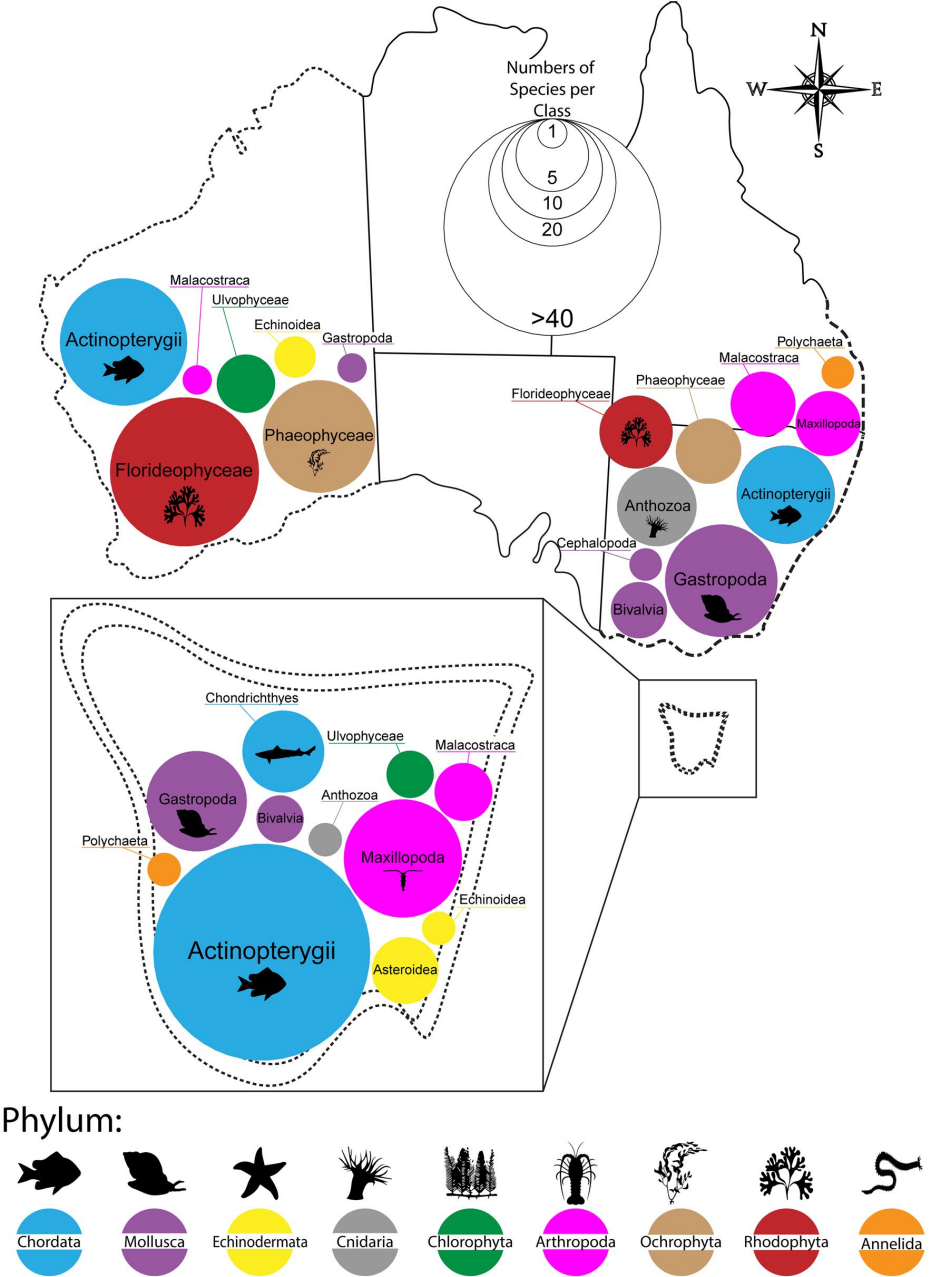
Species representation and spatial distribution of observed range shifting species across Australia. Circles represent number of species per class observed shifting by region (different border patterns) in Australia. Phyla are represented by different colours

Tasmania's coastal ocean is associated with more records of range shifting species than any other region of Australia's surrounding ocean (Figure 3; Table S4). This is likely to, in part, reflect that Tasmania is situated at the southernmost point of the Australian continental shelf and species are generally unable to viably extend their poleward distributions beyond this point due to physical habitat limitations. Historically, temperate reef communities off Tasmania have remained relatively stable (Stuart‐Smith et al., 2009). However, with an increasing number of novel species observed in this region and the concurrent decline in key habitat‐forming kelp forests, native ecosystems are undergoing rapid change (Ling, 2008). The geographical characteristic of Tasmania's coastal ocean has led to an accumulation of novel species assemblages among this region's native species (Johnson et al., 2011; Last et al., 2010). As native coastal species are unable to redistribute further polewards, Tasmania thus represents a ‘species sink’ as the rate of novel species arrivals is likely to outpace the rate of local extirpations/extinctions. Indeed, 48 fish species have been observed shifting their distribution in the Tasmanian region. This accounts for 64% of Australian marine fishes observed to be undergoing climate‐driven range shifts (Table S4). Of these, nearly all (45 of 48 fish species) are extending their southern range edge poleward, with only a small proportion of fishes observed contracting their equatorward range edges (2 of 48 fish species) or shifting both range edges poleward (1 of 48 fish species). Similarly, benthic invertebrates and algae in Tasmania are displaying a parallel trend with most species exhibiting distributional changes that are consistent with the predicted effects of climate change (Figure 5). However, to date only one fish is known to have become extinct over the recent decades—largely due to habitat loss (Last et al., 2020). Furthermore, the marine environment off south‐east Australia is warming at a rate that is more rapid than other regions of Australia's coastal ocean (Hobday & Pecl, 2014), which is likely to be driving a greater number of species redistributions relative to regions experiencing slower rates of ocean warming. Range shift studies from Tasmania also benefit from the availability of long‐term historical records, both from commercial fisheries (Last et al., 2010) and established biodiversity surveys (Pitt et al., 2010). These records offer baseline information on a greater number of species (specifically fish and invertebrates) relative to other regions.

**FIGURE 4:**
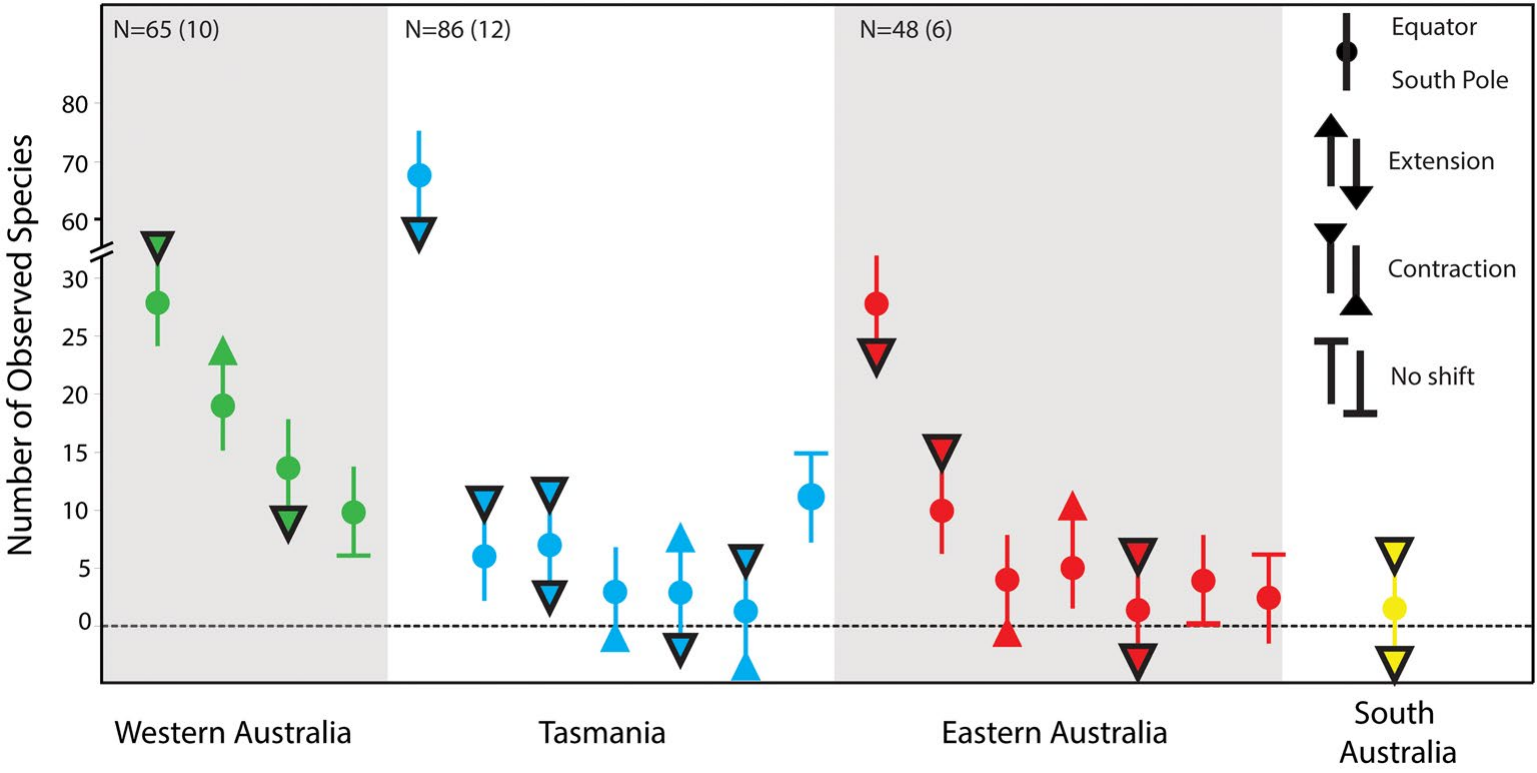
Each dot represents the number of species observed undergoing a specific range shift as well as the diversity of observed shifts in the literature. Arrows (or bar) represent both the range edge observed (equatorward or poleward), as well as the direction of the shift (extension, contraction or no observed shift). Arrows outlined in black denote the climate‐driven species redistributions that are consistent with the environmental effects of climate change. Please note that the y‐axis is not continuous, as the number of observations of poleward extensions in Tasmania are more than double the next greatest category (Western Australia poleward contractions). The total number of species shifting per region are indicated at the top and the number of species not observed shifting are in parentheses

While our review identified that the greatest number of species have been observed shifting in Tasmania's coastal ocean, the greatest number of publications documenting climate‐driven range shifts in marine species were from Western Australia's coastline (16 of 33 observation‐based Australian marine range shift publications). Much of this research was undertaken following Western Australia's 2011 marine heatwave event (Wernberg et al., 2012), with 81.3% (13 of 16 Western Australia publications) focusing on prolonged impacts following this extreme event. The persistence of anomalously warm sea surface temperatures during this marine heatwave facilitated overwintering of some sub‐tropical and tropical species in regions poleward of their historical distribution limits (Lenanton et al., 2017; Pearce et al., 2016). Concomitantly, temperate species (particularly algae) experienced significant die‐offs, resulting in the contraction of species equatorward range edges (Wernberg et al., 2016). Changes in the distributions of algae have been documented in greater numbers along Western Australia's coastline than from the rest of Australia combined (Figure 4), with 37 of 51 (~70%) shifts in algal species being detected within the region (Wernberg et al., 2011). These shifts in the distributions of habitat‐forming species off Western Australia have underpinned significant alterations to ecosystem structure and function (Wernberg et al., 2011, 2012, 2016).

The south‐eastern coast of Australia is the most populated region on the continent and encompasses both sub‐tropical to temperate climates. Marine range shift research undertaken off eastern Australia is characterized by the poleward extension of tropical species into sub‐tropical and temperate marine environments. For example, two‐thirds of publications from this region focused on tropical species, with a strong species bias towards invertebrates and intertidal community shifts (Caswell et al., 2020; Poloczanska et al., 2011). Additionally, there is a long history of tropical fish species recruiting to temperate habitats in this region, but historically very few survive over winter (Booth et al., 2011). However, tropical species have now been recorded overwintering in temperate ecosystems beyond their historical poleward range edges and have begun to establish populations in areas where minimum annual temperatures had previously prevented their persistence (Figueira & Booth, 2010). The tropicalization of Australia's south‐eastern coastline, much like Western Australia, is concerning given that some range extending species have been recorded degrading native communities (Vergés et al., 2016, 2019).

Concomitant with geographical biases, documented shifts in species distributions are not representative of the available ecosystems and climate regions across Australia. Specifically, despite Australia's coastlines extending from the equatorial tropics to high latitude temperate zones, there is a significant bias towards observations of species that have an affinity for temperate waters. For example, 152 of all 198 species reported to have displayed range shifts originate from temperate waters while tropical species accounted for 20.5% of observed range shifts (15 fish and 26 invertebrates out of 198 species total). This could reflect several different trends. First, it could be possible that a greater number of temperate species are undergoing range shifts due to a greater relative exposure to warming occurring off south‐western and south‐eastern Australia coastlines (Hobday & Pecl, 2014). Second, if contractions in the equatorward edges of the distributions of Australian tropical species are occurring, these may be falling outside the geographical extent investigated in numerous studies, including this review (i.e. outside of Australian waters). Indeed, no equatorward range edge contractions have been observed in Australian tropical marine species to date. An additional factor contributing to relatively few numbers of tropical species identified undergoing range changes is the lack of suitable available physical habitat in sub‐tropical and temperate Australian marine systems, even if suitable thermal conditions exist (Nay et al., 2020). For example, ~10% of coral reef fishes are obligate corallivores and up to 70% more are highly dependent on physical coral structure (Munday et al., 2008). As such, in the absence of suitable habitat, species that depend on coral or coral‐like structures (e.g. artificial reefs) will likely be limited in their capacity to permanently establish extralimital populations.

Despite the bias towards temperate species, many tropical species, particularly juvenile fish are observed beyond their historical poleward distributions, but they fail to overwinter (Booth et al., 2007, 2011; Figueira & Booth, 2010). With the rapid rate of warming, it is likely that more tropical species will begin to permanently establish (Figueira & Booth, 2010). Regardless, rapid shifts in the distribution of marine species and communities are highly likely to impact the ecological, economic and cultural values of Australia's marine systems (Pecl et al., 2017; Pinsky et al., 2020), however the magnitude of these impacts remain poorly understood.

## 6 SPECIES TRAJECTORIES

**FIGURE 5:**
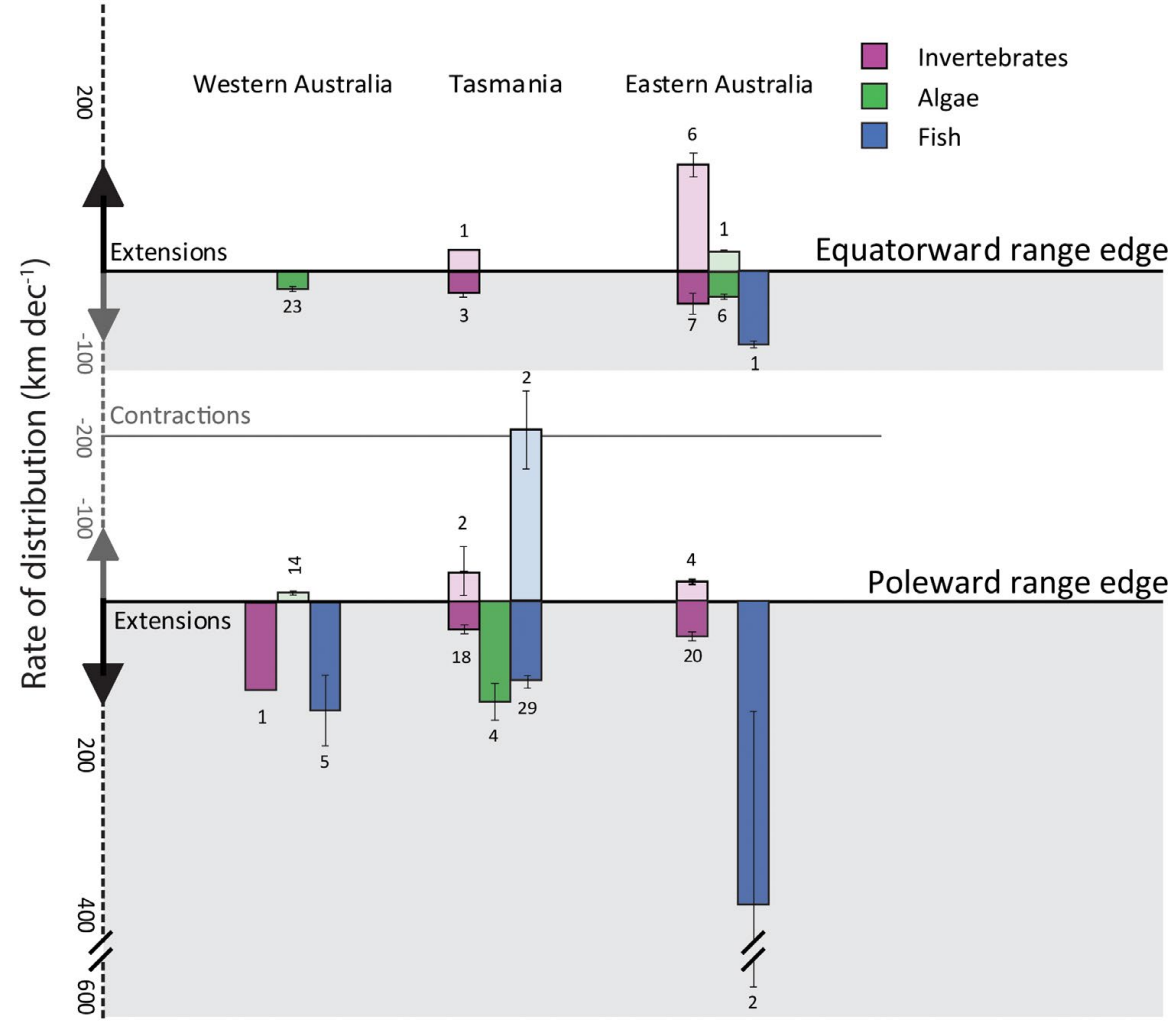
Bar graph depicting the rate of range shifts (km dec^−1^ ± SEM) of marine species across Australia both poleward, consistent with the environmental effects of climate change (solid bars within the shaded boxes) and equatorward (opaque bars). Colours represent different taxa: invertebrates (purple), algae (green) and fish. The numbers above/below each bar represent the number of species contributing to each bar. Please note that the y‐axis is not continuous

Poleward species redistributions are anticipated in response to the environmental effects of climate change (Parmesan & Yohe, 2003). The results of our literature analysis for Australian marine species are consistent with this expectation, with 87.3% (173 of 198 species) of species range shifts (exclusive of non‐shifting observations) occurring in a poleward direction (Figure 5). There are, however, some exceptions to the overwhelmingly poleward direction of Australian marine species redistributions. For example, 11.4% of observed range shifts (primarily algae and intertidal invertebrates) were in opposition to the expectation under climate change (see Poloczanska et al., 2011; Wernberg et al., 2011). The mechanisms underpinning these unanticipated changes in distribution are largely unknown, but could be associated with competitive release (Hawkins et al., 2008), human‐assisted dispersal (Cariton & Geller, 1993) or a result of historical fishing pressure on targeted marine species (Last et al., 2010).

Rates of redistribution (e.g. km per decade) and changes in range size (e.g. area occupied) have been the key quantitative descriptors utilized in studies observing and predicting range shifts in Australian marine taxa. For example, predicted rates of redistribution among coastal‐pelagic fishes off Australia have been found to range between 20 km (Robinson, Hobday, et al., 2015) and 108.8 km per decade (Champion et al., 2018), while the distributions of habitat‐forming kelps have been estimated to contract by 78% by 2100 relative to the present‐day (Martínez et al., 2018). Despite the importance of these metrics, there are many confounding factors that impede robust calculations of species rates of change. Indeed, within Australia, rates at which species are shifting are highly variable by taxa, region and shift trajectory (Figure 5; Tables S2 and S5). In addition, nearly all observed shifts in Australia are centred around changes occurring at species’ range edges. However, studies predicting species redistributions assessed numerous metrics of change, including shifts in the centroid of species distributions (8 of 16 studies), changes in the total area occupied (Martínez et al., 2018), shifts in phenology (Jacups, 2010) and changes in the latitudinal location of range edges (Hyndes et al., 2016; Table S1). Comparisons among rates of redistributions and changes in range size are desirable for assessing the relative sensitivity of Australian marine species to climate change; however, methodological differences among studies can undermine robust comparisons (Brown et al., 2016).

Research efforts have largely focused on quantifying spatial changes in only one range edge of species distributions. For example, of the ~200 marine species documented to be undergoing climate‐driven range shifts around Australia, range shifts in only 14 species (or 7%) have been documented at both the equatorward and poleward range edge, and of these, only four species (*Austrocochlea constricta*, *Patiriella exigua*, *Phyllospora comosa* and *Xenostrobus pulex*) are captured within the scope of a single study (see Pitt et al., 2010; Wernberg et al., 2011). This is because it is common for studies to have a local or regional (e.g. Tasmania, Eastern Australia, Western Australia) geographical focus that does not capture species complete geographical distributions.

Reports of species not undergoing range shifts within analyses are relatively limited. This may be associated with publication biases towards reporting the results of identified species redistributions (Csada et al., 1996; Hedges & Gurevitch, 1999; Przeslawski et al., 2012). Within our literature review database, five studies, out of 33 (15.1%), explicitly reported species (*n* = 29) that had been studied but identified not to be range shifting. Information regarding species that have not been found to be undergoing a climate‐driven redistribution can be just as important to identified range shifts, particularly for identifying species traits—physiological, morphological, behavioural or ecological—that hinder or facilitate species redistributions (Estrada et al., 2016; Pacifici et al., 2017; Sunday et al., 2015). The identification of species traits that are sensitive to climate‐driven environmental change may provide an opportunity for developing relative sensitivity rankings of species within climate change vulnerability assessments (Foden et al., 2019; Pecl, Ward, et al., 2014).

In addition to latitudinal shifts in species distributions, warming ocean temperatures have been shown to drive vertical redistributions of marine species to deeper depths (Nye et al., 2009). However, detecting the redistribution of species to greater depth can be challenging due to logistical constraints associated with recreational and scientific diving depth limitations (Fetterplace et al., 2018). Changes in sampling methodologies, such as the use of baited remote underwater monitoring systems (BRUVs) and other autonomous devices, offer the potential to facilitate monitoring of species ranges across a gradient of depth (Fetterplace et al., 2018; Giraldo‐Ospina et al., 2020). For example, BRUVs have already been applied to document species (within their latitudinal distribution) inhabiting waters deeper than previously known (Fetterplace et al., 2018). Similarly, following the 2011 Western Australian marine heatwave event, autonomous underwater vehicles conducted benthic surveys on deeper reefs (>30 m deep), revealing a buffering effect of depth on species whose abundances had declined in shallow warmer waters (Giraldo‐Ospina et al., 2020). Emerging technologies for monitoring ecological assemblages at depths greater than ~20 m have the potential to disentangle latitudinal and vertical shifts in the distributions of Australian marine biota in response to climate warming.

## 7 SYNTHESIS OF KNOWLEDGE GAPS AND FUTURE DIRECTIONS

Our review has highlighted evidence of extensive changes in the distribution of marine species around the Australian coastline. Nonetheless, given that huge regions of the Australian coastline are sparsely populated and have received little to no dedicated ecological monitoring, the number of marine species undergoing range shifts reviewed herein is almost certainly a considerable underestimate. Tropical systems and tropical species are in general woefully under‐explored, with observational evidence of species redistributions from sub‐tropical and temperate regions biased towards either algae, gastropods or fish. Predictions of potential future shifts are far fewer in number compared with the number of species already observed to be shifting and are largely biased towards coastal‐pelagic fishes. Moreover, only 2% of species observed to have undergone recent changes in distribution have had potential changes at both equatorward and poleward range limits explored within the one study—the vast majority of studies have focussed only on range shifts at the cooler poleward range limit.

Given that environmental changes in Australia's marine systems are almost certain to increase with ongoing climate change (Hobday & Lough, 2011), we note the need for future research to utilize standardized methods for documenting and predicting marine range shifts (e.g. Twiname et al., 2020) to improve comparability and assessments of species relative vulnerability. Such analyses could be characterized by descriptions and predictions of species distributions that use consistent time‐series and indices of species distributions (e.g. areas occupied or trailing range edges), while ensuring that environmental covariates for future projection studies are derived from consistent global circulation models forced under the same emissions scenarios. Many studies reviewed herein were hampered by the lack of baseline distributional information, and where there were historical observations these were often characterized by sampling bias and unequal spatiotemporal sampling effort. Rigorous and standardized biodiversity surveys are urgently required to provide the baseline information needed to assess future changes in species distributions. Rates of warming around most of the Australian coastline are greater than the global average and thus rates of species redistribution are likely to accelerate, with implications for Indigenous, commercial and recreational fisheries, as well as conservation and human health (Pecl et al., 2017).

## 8 GLOBAL LESSONS

Recently, there have been a large number of studies documenting climate‐driven changes in species distributions and exploring the effects on individual species and ecological communities worldwide (Bonebrake et al., 2017). The implications of large‐scale species redistribution for natural systems and human societies are considerable (Pecl et al., 2017), and there is an urgent need to improve our understanding of the current and future changes in biodiversity, and the complex processes underpinning these (Twiname et al., 2020), to provide the best possible support for current and future management and adaptation efforts. Here, we provide a high‐level summary of key points emerging from our synthesis of Australian marine range shift studies that are relevant globally:

- The development of standardized protocols for applying methods to quantify range shifts will facilitate greater comparability among studies and improved accuracy of global metanalyses qualifying, for example, rates of marine species distributions.
- Where possible studies need to encompass complete distributions of species (i.e. examine potential changes in range limits at equatorward and poleward range limits), and also report non‐shifting species to allow the development of a more complete picture and advance trait‐based assessments of species redistributions.
- The ideal data to document changes in species distributions are rigorous, structured surveys repeated over time and these should be facilitated wherever possible. However, ‘perfect is the enemy of good’ and useful insights can be generated by careful compilations of varied data.
- Citizen Science has been increasingly highlighted in the literature and is a valuable approach for observing a wide range of species. Furthermore, engaging fishers, divers, marine naturalists, boaters and other marine users in the documentation of changes in the marine environment has the dual advantage of providing data and actively engaging marine communities on the biological effects of climate change simultaneously (Nursey‐Bray et al., 2018).

​

Lastly, adaptation to climate change should happen concurrently and not wait for climate change impact research to justify action. Marine‐dependent individuals, organizations and user‐groups in fast‐changing regions of the world are already adjusting their behaviour to accommodate changes. For example, divers and fishers from south‐eastern Australia are undertaking autonomous behavioural adaptations to adjust the spatial and temporal timing of their activities in response to climate‐driven species redistributions (Pecl, Ogier, et al., 2019). However, these are generally reactive forms of adaptation, and collaboration between private and public sectors is essential for developing anticipatory strategies for managing marine range shifts, which may be guided by the paradigm of whether to persecute, protect or ignore species arriving in novel environments (Scheffers & Pecl, 2019).

## Supporting information

Table S1‐S5

## Data Availability

The data that support the findings of this study are available from the corresponding author upon reasonable request.
